# Molnupiravir—A Novel Oral Anti-SARS-CoV-2 Agent

**DOI:** 10.3390/antibiotics10111294

**Published:** 2021-10-23

**Authors:** Ching-Chi Lee, Chih-Chia Hsieh, Wen-Chien Ko

**Affiliations:** 1 Clinical Medicine Research Center, National Cheng Kung University Hospital, College of Medicine, National Cheng Kung University, Tainan 704, Taiwan; chichingbm85@gmail.com; 2 Department of Internal Medicine, National Cheng Kung University Hospital, College of Medicine, National Cheng Kung University, Tainan 704, Taiwan; 3 Department of Emergency Medicine, National Cheng Kung University Hospital, College of Medicine, National Cheng Kung University, Tainan 704, Taiwan; hsiehchihchia@gmail.com; 4 Department of Medicine, College of Medicine, National Cheng Kung University, Tainan 701, Taiwan

**Keywords:** COVID-19, antiviral agents, molnupiravir, RNA polymerase inhibitor, clinical trial

## Abstract

Since December 2019, severe acute respiratory syndrome coronavirus 2 (SARS-CoV-2) has rapidly resulted in a global pandemic with approximately 4 million deaths. Effective oral antiviral agents are urgently needed to treat coronavirus disease-2019 (COVID-19), block SARS-CoV-2 transmission, and prevent progression to severe illness. Molnupiravir (formerly EIDD-2801), a prodrug of beta-d-N4-hydroxycytidine (EIDD-1931) and an inhibitor of RNA-dependent RNA polymerase, possesses significant activity against SARS-CoV-2. Its prophylactic efficacy has been evidenced in a ferret model. Two phase-I trials (NCT04392219 and NCT04746183) have demonstrated that oral molnupiravir is safe and well-tolerated at therapeutic doses. After five-days of oral molnupiravir therapy, satisfactory efficacies, assessed by eliminating nasopharyngeal virus in patients with early and mild COVID-19, were disclosed in two phase-II trials (NCT04405739 and NCT 04405570). Two phase-II/III trials, NCT04575597 and NCT04575584, with estimated enrollments of 1850 and 304 cases, respectively, are ongoing. The NCT04575597 recently released that molnupiravir significantly reduced the risk of hospitalization or death in adults experiencing mild or moderate COVID-19. To benefit individual and public health, clinical applications of molnupiravir to promptly treat COVID-19 patients and prevent SARS-CoV-2 transmission may be expected.

## 1. Introduction

Coronaviruses, single-stranded, positive-sense, and enveloped RNA viruses, are named based on the appearance of a solar corona under an electron microscope [1] and have the ability to cause zoonosis [2]. In addition to severe acute respiratory syndrome coronavirus (SARS-CoV) and Middle East respiratory syndrome coronavirus (MERS-CoV) [3,4], the third coronavirus, SARS-CoV-2, causing the global pandemic of coronavirus disease 2019 (COVID-19), was first recognized in December 2019 in China [5]. To date, more than 186 million cases of COVID-19 and 4.0 million deaths have been reported globally [6]. Following major transmission by droplets, clinical manifestations of COVID-19 range widely from asymptomatic infections to life-threatening diseases [7,8]. Severe COVID-19 is associated with fatality in older patients, immunocompromised individuals, and those with comorbid hypertension, diabetes, malignancies, cardiovascular diseases, or chronic lung diseases [8,9,10].

The development of effective and easily administered anti-SARS-CoV-2 agents is urgently anticipated to reduce viral load and transmission, disease severity, hospitalization, and even deaths. Antiviral agents under investigation include viral fusion inhibitors [11], inhibitors of viral RNA-dependent RNA polymerase (RdRp) [12], and inhibitors of viral protein synthesis [13]. RdRP is a preferred target for drug repurposing, because of its specific domain and lack of counterparts in human cells [14]. Notably, the most promising, broad-spectrum class of viral RdRp inhibitors is the analog of nucleoside or nucleotide, including remdesivir, molnupiravir, favipiravir, galidesivir, ribavirin, sofosbuvir, and tenofovir [12]. Of the numerous analogs of nucleoside or nucleotide, the previously established evidence only indicated that remdesivir successfully improves the clinical outcomes of hospitalized patients with COVID-19, with a significantly reduced period to clinical recovery [15]. However, its clinical application appears to have two vast limitations: only focusing on less critically ill patients and administering by the intravenous route. Accordingly, the intent of this report is to provide a detailed overview of molnupiravir, an oral and novel RdRp inhibitor.

## 2. Molnupiravir

**Figure 1:**
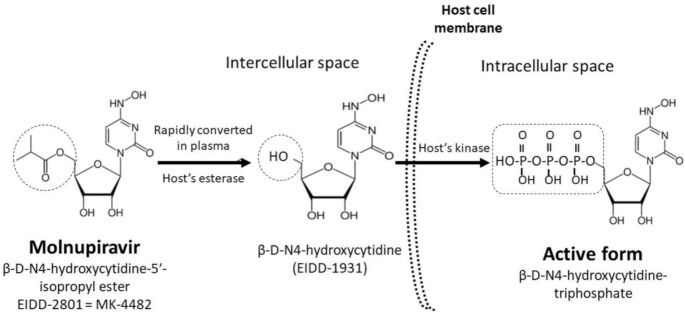
In plasma, molnupiravir is rapidly converted to EIDD-1931, which is converted into EIDD-1931-triphosphate, the active antiviral compound, after penetration into SARS-CoV-2-infected cells.

Beta-d-N4-hydroxycytidine (Emory Institute for Drug Development [EIDD]-1931) is an orally bioavailable ribonucleoside analog (Figure 1) and has broad-spectrum activity against numerous RNA viruses in animal models [16,17,18,19]. Molnupiravir, β-d-N4-hydroxycytidine-5′-isopropyl ester (EIDD-2801), is a prodrug of β-d-N4-hydroxycytidine (EIDD-1931) and is rapidly converted into EIDD-1931 in the plasma by the host’s esterase [20,21]. After entering host cells, EIDD-1931 is intracellularly transformed into its active form, β-d-N4-hydroxycytidine-triphosphate (Figure 1), which inhibits viral replication through its incorporation into the viral genome. Consequently, the accumulation of mutations results in the viral error catastrophe [22].

The recognized pharmacodynamic properties of molnupiravir are summarized in Table 1. Based on previous studies emphasizing both in vitro and in vivo efficacies against coronaviruses and the good bioavailability in animal models [23,24], molnupiravir was first regarded as an oral and direct-acting anti-SARS-CoV-2 agent.

### 2.1. Animal Models

In a humanized mouse model (i.e., human lung-only mice), the therapeutic and prophylactic administration of molnupiravir markedly reduced the in vivo replication and pathogenesis of SARS-CoV-2 in type-2 pneumocytes [25]. In addition, molnupiravir therapy completely blocked SARS-CoV-2 transmission to untreated animals in a ferret model. We believe that early molnupiravir treatment in asymptomatic or mildly symptomatic patients can potentially prevent viral transmissions among susceptible individuals because the prophylactic efficacy of oral molnupiravir was evidenced in vivo [26].

In another mouse model, molnupiravir significantly exhibited antiviral activity when administered 2 hours before, or 12 or 24 h after SARS-CoV-2 infections (as evidenced by a decline in the pulmonary viral load) and clinical success (as indicated by the returned body weight and improved pulmonary function) during the five-day observation [24]. As a result, molnupiravir has been shown to be effective against SARS-CoV-2 infections with variants of concern, B.1.1.7 or B.1.351 in hamsters, which was supported by a reduction in the pulmonary viral load, regained body weight, and reduction of disease severity [27].

### 2.2. Resistant Barrier

In an in vivo study, low-level resistance against EIDD-2801 was difficult to achieve because of the need for multiple transition mutations across the coronavirus genome [19]. Although EIDD-2801-driven mutagenesis correlates significantly with reductions in viral load, which are strongly suggestive of an error catastrophe-driven mechanism of therapeutic antiviral action, EIDD-2801 did not increase mutations in host cellular RNA under a therapeutic dosage [28].

**Table 1:** Detailed information on molnupiravir.

**Compound Name**	β-d-N4-hydroxycytidine-5′-isopropyl ester (EIDD-2801)
Active form	β-d-N4-hydroxycytidine-triphosphate
Molecular weight	329.31 Da
**Classification of Antiviral Agents**	Inhibitor of RNA-dependent RNA polymerase
Antiviral mechanism [20,21]	Inhibits viral replication by incorporation into the viral genome and causes the accumulation of mutations
In vitro activity against virus types [16,17,18,19]	Coronaviruses, Venezuelan equine encephalitis virus, respiratory syncytial virus, Ebola virus, influenza A and B viruses, and Chikungunya virus
**Administration Route**	Oral
**Anti-SARS-CoV-2 Activity (Vero Cell Line) [24]**	
IC (inhibitory concentration)_50_	0.3 μM
CC (cytotoxic concentration)_50_	>10 μM
Selectivity index	>100
**Pharmacodynamic Properties (Single Dosing of 800 mg) [29]**	
AUC_last_ (mean)	8720 ng·h/mL
AUC_inf_ (mean)	8720 ng·h/mL
C_max_ (mean)	3640 ng/mL
t_max_	1.00 h
t_1/2_ (mean)	1.29 h
Ae_0–24_ (mean)	18.0 mg
Fe_0–24_ (mean)	2.86%
**Resistance**	1. High resistant barrier across the coronavirus genome [19]; 2. Low potential for mutations in cellular RNA of the host [28].
**Clinical Applications**	
Prophylactic efficacy	Evidenced in animal models [26]
Therapeutic efficacy	For patients with asymptomatic or mild severity of COVID-19, as evidenced by phase-I and II trials [30,31,32]

Ae_0–24_—the amount of the dose administered recovered in urine from time zero to 24 h postdosing; AUC_inf_—the area under plasma concentration-time curve from time zero extrapolated to infinity; AUC_last_—the area under plasma concentration-time curve from time zero to the last measurable nonzero concentration; Cmax—maximum observed concentration; Fe_0–24_—the percentage of the dose administered recovered in urine from time zero to 24 h postdosing; t_1/2_—terminal elimination half-life; t_max_—the time of the maximum observed concentration.

## 3. Clinical Trials of Molnupiravir

### 3.1. Phase I

Two completed phase-I trials have been reported recently (Table 2). The first trial, NCT04392219, aimed to investigate the safety, tolerability, and pharmacokinetics of molnupiravir in healthy volunteers [29]. Focusing on adults with early symptomatic COVID-19, the optimal dose and safety of molnupiravir were evaluated through a multicenter, multistage, open-label, phase-Ib/IIa randomized trial (NCT04746183) [30].

In the first-in-human, randomized, double-blind, placebo-controlled trial (NCT04392219) [29], the single and multiple dosing of molnupiravir and the effect of food intake on pharmacokinetics in healthy volunteers were evaluated. After oral administration of molnupiravir, EIDD-1931 appeared rapidly in plasma and achieved the maximum serum concentration with a median time of 1.00 to 1.75 h. The serum concentration slowly declined with a half-life of approximately 1 h, regardless of whether single or multiple doses were administered. In a dose-proportional manner, the mean maximum concentrations and the areas under the concentration versus time curves increased. Notably, the concern regarding the accumulation of serum concentrations following the administration of single or multiple doses was not observed.

When orally administered in fed participants, a decrease in the absorption rate without a decrease in overall exposure was evident [29]. Molnupiravir was well tolerated because fewer participants experienced adverse events. Unexpectedly, the incidence of adverse events in the subjects administered with placebo was higher. Among the participants administered with molnupiravir, only one discontinued treatment due to skin rash, and no serious adverse events were reported. No pathological or clinical abnormalities in laboratory data, vital signs, or electrocardiography were observed.

AGILE is a multicenter, multistage open-label, phase-Ib/IIa randomized platform for the rapid evaluation of suitable candidate agents for COVID-19 [33]. Through this platform, the optimal dose and safety of molnupiravir in 18 adults with COVID-19 diagnosed by reverse transcriptase polymerase chain reaction (RT-PCR), within five days of symptom onset, were evaluated in a phase-I trial (NCT04746183) [30]. Four of four participants, four of four participants, and one of four participants receiving 300, 600, and 800 mg of molnupiravir twice daily for 5 days, respectively, and five of six controls receiving placebo, experienced at least one adverse event, and all events were mild. Accordingly, molnupiravir was regarded as well tolerated at 400, 600, or 800 mg doses with no serious or severe adverse events, and thereby was recommended for further phase-II trials to determine an optimal dose. However, the evidence determining the 5-day period as the optimal course in this trail is limited.

### 3.2. Phase II

Two phase-II trials have been completed (Table 2). One enrolled hospitalized participants with COVID-19 aimed to assess the rate of viral clearance in nasopharyngeal sites as the efficacy of molnupiravir therapy (NCT04405739) [31]. This trial included 87 participants with COVID-19 diagnosed by RT-PCR who presented signs or symptoms within 7 days after onset of symptoms. Their median (range) period between COVID-19 onset and diagnoses was 4.62 days (1.40–7.54 days). Participants randomly received 200 mg of molnupiravir or placebo twice daily for 5 days; 52 participants received molnupiravir and the remainder received the placebo. The proportion of participants with SARS-CoV-2 growth on nasopharyngeal cultures on day 3 were 20.4% and 28% in the molnupiravir and placebo groups, respectively (*p* = 0.56). Conversely, placebo-treated participants remained a significantly higher percentage of SARS-CoV-2 growth on cultures on day 5 (24% vs. 0%; *p* = 0.001) than molnupiravir participants. Although the incidence of adverse effects in the participant and placebo groups was not disclosed, this trial documented the ability of oral short-course administration of molnupiravir in reducing the infectiousness of SARS-CoV-2.

The safety and efficacy between molnupiravir and placebo-treated participants were compared in another phase-II trial, in which the antiviral efficacy was determined by detecting viral clearance using RT-PCR (NCT04405570) [32]. Two hundred outpatient participants diagnosed with COVID-19 by RT-PCR and experienced symptom onset within 7 days were eligible. Participants were randomized at a ratio of 1:1 to 200 mg of molnupiravir or placebo, or a ratio of 3:1 to molnupiravir (400 or 800 mg) or placebo, twice daily for 5 days. Compared to placebo-treated participants, the proportion of detectable SARS-CoV-2 in nasopharyngeal swabs using RT-PCR was significantly lower (1/53, 1.9% vs. 9/54, 16.7%; *p* = 0.02) in participants receiving 800 mg of molnupiravir on day 3. On day 5, nasopharyngeal SARS-CoV-2 was not genetically recognized in any participants receiving 400 or 800 mg of molnupiravir (0/42 and 0/53, respectively), compared with 11.1% (6/54) of placebo groups (*p* = 0.03). Compared to placebo-treated participants, a shorter period (medians, 14 vs. 27 days; *p* = 0.013) of the time to viral clearance measured by RT-PCR in nasopharyngeal swabs among those administered 800 mg was disclosed. Likewise, molnupiravir was generally well tolerated with similar proportions of adverse events across all groups. Taking the most frequent event as an example, dizziness and insomnia accounted for 8.7% and 8.7% in the 200 mg group, 1.6% and 1.6% in the 400 mg group, 0% and 1.8% in the 800 mg group, and 0% and 6.5% in the placebo group, respectively.

In summary, these studies indicate that oral molnupiravir is highly effective in reducing nasopharyngeal SARS-CoV-2 and offers a favorable tolerability and safety profile. However, we consider it an issue that these endpoints are limited to virological clearance, noting that it lacks clinical value in the relief of lingering symptoms/signs and rebounding viral load, after short-course (i.e., 5-day) treatment.

### 3.3. Phase III

Two phase-III trials registered at ClinicalTrials.gov designed to evaluate the safety, tolerability, and efficacy of molnupiravir compared to placebo recruited 1850 non-hospitalized participants in one trial (NCT04575597) and 304 hospitalized participants in another (NCT04575584), as shown in Table 3 [34]. Their primary hypothesis is that molnupiravir is superior to placebo by assessing the proportion of participants who are hospitalized and/or die within 29 days after administration.

In the NCT04575584 trial, molnupiravir or the placebo was administered within 7 days after the onset of symptoms or signs. However, we are concerned that this 7-day window makes it challenging to enroll participants in the real world. From our experience, patients usually observe themselves if their symptoms worsen for a couple of days before they decide to take on medical treatment. Therefore, we believe the longer window period is more suitable to enroll sufficient participants for analyses. Furthermore, in both phase-III trials, one of the efficacies is assessed by the proportion of un-hospitalized participants. We also consider the indication diversity of hospitalization in different study areas might result in the outcome-reporting bias.

In early October 2021, Ridgeback Biotherapeutics first announced the preliminary result in the trial (NCT04575597), in which molnupiravir significantly reduced the risk of hospitalization or death in non-hospitalized adults experiencing mild or moderate COVID-19 [35]. By the interim analysis, 7.3% of patients who received molnupiravir had significantly lower rates of hospitalization or mortality within 29 days following randomization (28/385, 7.3% vs. 53/377, 14.1%; *p* = 0.001), compared to the placebo-treated patients. Moreover, through day 29, no death was observed in molnupiravir-treated patients, compared to eight deaths in the placebo group. Notably, the similar incidence of overall (35% and 40%, respectively) and drug-related (12% and 11%, respectively) adverse effects was disclosed in the molnupiravir and placebo groups. Fewer subject proportions of discontinued treatment due to adverse events were observed in the molnupiravir (1.3%) and placebo (3.4%) groups.

## 4. Conclusions

Molnupiravir is an oral, direct-acting agent with in vivo activity against SARS-CoV-2 and can successfully treat infected animals. Moreover, molnupiravir was found to be highly effective at reducing the nasopharyngeal viral load and had a favorable safety and tolerability profile in COVID-19 patients receiving short-course, five-day therapy. Notably, an updated phase-III trial revealed that molnupiravir significantly reduced the risk of hospitalization or death in adults experiencing mild or moderate COVID-19. To benefit individual and public health, the clinical role of oral molnupiravir in the early treatment of patients experiencing asymptomatic or mild COVID-19 and the prevention of SARS-CoV-2 transmission will become more evident in the near future.

## Figures and Tables

**Table 2:** Completed clinical phase-I and II trials of molnupiravir.

Phase	Registered No.	Participants	Study Site	Study Design	Primary Aims	Principal Results	Reference
I	NCT04392219	64 healthy volunteers	United Kingdom	Randomized, double-blind, placebo-controlled, single-center	Effects of single or multiple doses and food intake on pharmacokinetics	Similar pharmacokinetics after the administration of single or multiple doses; limited effects of food intake on absorption	[29]
I	NCT04746183	18 adults within 5 days of COVID-19 symptom onset	United Kingdom	Dose-escalating, open-label, randomized-controlled, single-center	Safety and tolerability of multiple ascending doses to recommend a dose for the phase-II trial	Well tolerated at 400, 600, or 800 mg doses	[30]
II	NCT04405739	78 adults with onset of COVID-19 signs or symptoms within 7 days	United States	Double-blind, randomized, placebo-controlled, multicenter trial	Rates of viral clearance, by viral cultures in nasopharyngeal sites, as efficacies of molnupiravir	Compared to the placebo, the efficacies were significantly different on day 5, but not on day 3 after administration	[31]
II	NCT04405570	202 outpatients with onsets of COVID-19 symptoms within 7 days	United States	Double-blind, randomized, placebo-controlled, multicenter trial	Efficacies of molnupiravir based on the proportions of undetectable SARS-CoV-2 and alterations in the viral load in nasopharyngeal swabs detected using RT-PCR	Compared to the placebo, the effect appears significant on days 3 and 5 after administration	[32]

**Table 3:** Ongoing phase-III trials of molnupiravir.

Registered No.	Participants	Study Site	Study Design *	Primary Outcome *	Reference
NCT04575597	1850 non-hospitalized adults with mild or moderate COVID-19	The United States, Canada, Brazil, Mexico, Chile, Colombia, Japan, Taiwan, Philippines, Israel, Germany, France, Poland, Spain, Sweden, United Kingdom, Russian, Ukraine, South Africa (total 141 locations)	Double-blind, randomized-controlled, multicenter	1. Time-to-sustained recovery (up to 29 days); 2. Percentage of participants experiencing adverse events (up to 7 months); 3. Percentage of withdrawal participants due to adverse events (up to 6 days).	[34]
NCT04575584	304 hospitalized adults with mild, moderate, or severe COVID-19	The United States, Canada, Brazil, Mexico, Chile, Colombia, South Korea, Philippines, Israel, France, Poland, Spain, United Kingdom, Russian, Ukraine, South Africa (total 89 locations)			[34]

* The similarity of study design and primary outcome in two trials was noticed.

## Data Availability

Data are available from the corresponding authors on reasonable request.
